# Genome‐Wide Population Structure Analyses of Three Minor Millets: Kodo Millet, Little Millet, and Proso Millet

**DOI:** 10.3835/plantgenome2019.03.0021

**Published:** 2019-11-01

**Authors:** Matthew Johnson, Santosh Deshpande, Mani Vetriventhan, Hari D. Upadhyaya, Jason G. Wallace

**Affiliations:** ^1^ International Crops Research Institute for the Semi‐Arid Tropics (ICRISAT) Patancheru 502324 Telangana India; ^2^ Johnson Institute of Plant Breeding, Genetics, and Genomics Univ. of Georgia 111 Riverbend Rd. Athens GA

## Abstract

**Core Ideas:**

- Developed genome‐wide SNP marker data for kodo, proso, and little millet
- Marker data used to analyze genetic diversity
- Heritability results of various traits used to validate genetic data

Millets are a diverse group of small‐seeded grains that are rich in nutrients but have received relatively little advanced plant breeding research. Millets are important to smallholder farmers in Africa and Asia because of their short growing season, good stress tolerance, and high nutritional content. To advance the study and use of these species, we present genome‐wide marker datasets and population structure analyses for three minor millets: kodo millet (*Paspalum scrobiculatum* L.), little millet (*Panicum sumatrense* Roth), and proso millet (*Panicum miliaceum* L.).We generated genome‐wide marker data sets for 190 accessions of each species with genotyping‐by‐sequencing (GBS). After filtering, we retained between 161 and 165 accessions of each species, with 3461, 2245, and 1882 single‐nucleotide polymorphisms (SNPs) for kodo, little, and proso millet, respectively. Population genetic analysis revealed seven putative subpopulations of kodo millet and eight each of proso millet and little millet. To confirm the accuracy of this genetic data, we used public phenotype data on a subset of these accessions to estimate the heritability of various agronomically relevant phenotypes. Heritability values largely agree with the prior expectation for each phenotype, indicating that these SNPs provide an accurate genome‐wide sample of genetic variation. These data represent one of first genome‐wide population genetics analyses—and the most extensive—in these species and the first genomic analyses of any sort for little millet and kodo millet. These data will be a valuable resource for researchers and breeders trying to improve these crops for smallholder farmers.

**Abbreviations**
GBS: genotyping‐by‐sequencing
ICRISAT: International Crops Research Institute for the Semi‐Arid Tropics
SNP: single‐nucleotide polymorphism
SSR: simple‐sequence repeat

It is estimated that there are up to 7000 cultivated crop species in the world (Khoshbakht and Hammer 2008), yet major breeding and research efforts have focused on just a small number of these (Hammer et al., 2001). Many crops that have been ignored by modern research are still essential to the local communities that have relied on them for thousands of years, and they have potential for diversifying cropping systems around the world (Naylor et al., 2004). These crops are also genetic resources to increase global food security as climate changes and resources (land, water, fertilizers) become more limited. The ability to generate inexpensive, genome‐wide data can quickly bring some of these crops into the modern genomics era (Varshney et al., 2012).

Millets are a diverse group of small‐seeded grains that have been largely overlooked by modern genetics research. Although pearl millet [*Pennisetum glaucum* (L.) R. Br. syn. *Cenchrus americanus* (L.) Morrone], foxtail millet [*Setaria italica* (L.) P. Beauv.], and finger millet [*Eleusine coracana* (L.) Gaertn.] all have complete genome sequences and well‐established germplasm resources (Varshney et al., 2017; Zhang et al., 2012; Prasad 2017; Sehgal et al., 2015; Hittalmani et al., 2017; Jia et al., 2013; Bennetzen et al., 2012; Hatakeyama et al., 2017), most other millets have few if any resources available (Goron and Raizada, 2015). These crops are often important for smallholder farmers, especially in southeastern Asia and sub‐Saharan Africa (FAO; http://www.fao.org/docrep/W1808E/w1808e0e.htm).

Proso millet, little millet, and kodo millet are three minor millets with limited modern genetic resources (Table 1), although proso millet has recently seen a significant increase in genomic studies (e.g., Rajput and Santra [2016], Habiyaremye et al. [2017], and Yue et al., [2016]), including a reference genome (Zou et al., 2019). Various germplasm repositories maintain collections of these species (Goron and Raizada, 2015). The current study focuses on those held by the International Crops Research Institute for the Semi‐Arid Tropics (ICRISAT), which maintains collections with 849, 473, and 665 accessions of proso, little, and kodo millet, respectively. These collections have been assessed for morphological and agronomic traits, and representative core collections have been created for each of them (Upadhyaya et al., 2011,^2014^). These millets are hardy C_4_ grasses (Upadhyaya et al. 2014; Brown, 1999) with nutritional content on par with or superior to the major grains (Vetriventhan and Upadhyaya, 2018; Mengesha, 1966; Saleh et al., 2013; Kalinova and Moudry 2006).

### Proso Millet

Proso millet is believed to have been domesticated ∼10,000 yr ago. There have been multiple centers of origins proposed: northwestern China (Bettinger et al., 2010a, 2007,b), central China (Lu et al., 2009), and inner Mongolia (Zhao, 2005), with the most recent evidence pointing toward either one domestication event in China or one in China and one in Europe (Hunt et al., 2011). It is the third‐oldest cultivated cereal after wheat (*Triticum aestivum* L.) and barley (*Hordeum vulgare* L.) (Habiyaremye et al., 2017; Upadhyaya et al., 2011). Proso millet is valued for its low water requirements (330 mm) and short growing season (60 d) (Habiyaremye et al., 2017; Shanahan et al., 1988). Proso millet varieties are classified into five races based on inflorescence morphology: miliaceum, patentissimum, contractum, compactum, and ovatum (de Wet et al., 1983). Proso millet is tetraploid (2*n* = 4*x* = 36) (Saha et al., 2016) with an allotetraploid origin (Hunt et al., 2014). Proso millet has historically be the most widely grown of the three millets studied here, with cultivation concentrated in the former Soviet Union and India (Roshevits, 1980) and with significant production in the United States (Baltensperger, 2002).

**Table 1:** Genetic resources: Total ICRISAT accessions (http://genebank.icrisat.org/Default, accessed November 2018); proso millet core collection (Upadhyaya et al., 2011), kodo and little millet (Upadhyaya et al., 2014); ploidy information (Upadhyaya et al., 2011,^2014^); and estimated genome size for proso millet (Kubesova et al., 2010).

Species	Total ICRISAT accessions	Accessions in ICRISAT core collection	Expressions sequence tags	Complete coding sequences	Ploidy level	Estimated genome size
						Mbp
Proso millet	849	106	195	7	2*n* = 4*x* = 36	1020.5
Kodo millet	665	75	29	0	2*n* = 4*x* = 40	unknown
Little millet	473	55	12	0	2*n* = 4*x* = 36	unknown

Proso millet genetic diversity has been investigated with a variety of genetic markers; however, most of them at a very small scale (<100 markers; reviewed in Habiyaremye et al. [2017]). Recently, however, the most extensive genetic analysis in proso millet identified over 400,000 SNP markers and 35,000 simple‐sequence repeats (SSRs) from the transcriptomes of two proso millet accessions (Yue et al., 2016). Population‐level analyses were made by Rajput and Santra (2016) using 100 SSRs and 90 proso millet accessions; it was found that there were some connections between genetic clustering and geographic origin. There also has been quantitative trait loci mapping using 833 SNPs from GBS data (Rajput and Santra, 2016) and a just‐published reference genome (Zou et al., 2019).

### Kodo Millet

Kodo millet was domesticated in India ∼3000 yr ago, and India has historically been the major center of cultivation (de Wet et al., 1983). Kodo millet accessions have been classified into three races based on panicle morphology: regularis, irregularis, and variabilis (de Wet et al., 1983; Prasada Rao et al., 1993). Kodo millet is tetraploid (2*n* = 4*x* = 40) (Saha et al., 2016) and it is valued for its ability to produce consistently in hot, drought‐prone arid and semiarid land (Dwivedi et al., 2012). A few sets of molecular markers have been developed for kodo millet based on random‐amplification‐of‐polymorphic‐DNA (RAPD) markers (M'Ribu and Hilu 1996), gene‐specific primer sets (Kushwaha et al., 2014), and semitargeted polymerase chain reaction amplification (Yadav et al., 2016); the latter study concluded that the 96 accessions they used could be divided into four groups that showed little connection between geographic region and genetic relationship of the accessions. There have been no truly genome‐wide datasets on this species before now.

### Little Millet

Little millet was domesticated ∼5000 yr ago in India (de Wet et al., 1983). It has historically been grown mainly in India, Mayanmar, Nepal, and Sri Lanka (Prasada Rao et al., 1993). Little millet accessions have been classified into two races based on panicle morphology, nana and robusta, with two subraces per race (laxa and erecta for nana and laxa and compacta for robusta) (de Wet et al., 1983; Prasada Rao et al., 1993). Little millet is tetraploid (2*n* = 4*x* = 36) (Saha et al., 2016). Like kodo millet, little millet can give consistent yields on marginal lands in drought‐prone arid and semiarid regions, and it is an important crop for regional food stability (Dwivedi et al., 2012). Little millet is arguably the least studied of these three millets, and we are unaware of any molecular markers developed for it outside of specific single genes (Goron and Raizada 2015) and a small set of RAPD markers whose details were not described (M.S. Swaminathan Research Foundation, 2000).

### Expanding Genetic Resources

As mentioned above, the genetic and genomic resources of proso millet, kodo millet, and little millet are very limited (Saha et al., 2016; Goron and Raizada 2015), although the situation for proso millet, at least, is improving. The main focus of the resources have been centered on the accessions stored in gene banks, expression sequence tags, and complete coding sequences.

Core collections for each of these species were created several years ago consisting of ∼10% of ICRISAT's collection for each species (Upadhyaya et al., 2011,^2014^). These collections have been assessed for morphoagronomic traits but no genetic data has been available for them. Our goal in this project was to generate genome‐wide marker data on each of these species to enable population genetic analysis and empower breeders and researchers to make more informed decisions about germplasm selection and representation. We genotyped 190 accessions of each species, including the entire core collections of each using GBS (Elshire et al., 2011) to generate the most comprehensive population genetics resource for each of these species to date. These data dramatically expand the genomic resources available to each of these crops and will help make better use of them moving forward.

## MATERIALS AND METHODS

### Plant Materials

One‐hundred ninety accessions (Supplemental Data) were taken from the ICRISAT genebank for each millet species. These included the accessions from the core collection for proso millet, kodo millet, and little millet (Upadhyaya et al., 2011,^2014^). The remaining samples were chosen to broadly sample the available diversity based on the cluster information used to create the core collections.

### DNA Extraction and Sequencing

Seedlings of each accession were grown at the ICRISAT research station in Patancheru, India, in 2015. DNA was extracted from 55‐d‐old plants, with one plant from each accession used for extraction, by the modified CTAB method (Mace et al., 2003), lyophilized, and shipped to the Genomic Diversity Facility at Cornell University for GBS (Elshire et al., 2011). The GBS library preparation followed standard methods (Wallace and Mitchell 2017) using the PstI restriction enzyme. For kodo millet, 190 samples plus two blanks were multiplexed into a single lane for sequencing on an Illumina HiSeq 2500 with single‐end 100‐bp sequencing. For little and proso millet, the procedure was similar except that samples were multiplexed into two lanes of 95 samples plus one blank. The raw sequence data for these samples is available at the Sequence Read Archive, accessions PRJNA494158.

### Single‐Nucleotide Polymorphism Calling

Single‐nucleotide polymorphisms were called from raw sequencing data using the TASSEL (Bradbury et al., 2007) GBS v2 SNP pipeline. Since this pipeline uses alignment to a reference genome and none of these species had such a reference when we performed the analyses, we included slight alterations to make use of the UNEAK filter (Lu et al., 2013) for reference‐free alignment of tags to each other. All code for this analysis is available in the Supplemental data files and on GitHub (https://github.com/wallacelab/2018_minor_millets).

### Filtering

Low‐quality SNPs were removed by filtering the raw genotype data to remove sites with >50% missing data, minor allele frequency <5%, and >20 to 25% heterozygosity. (The heterozygosity filter removes false SNPs as a result of paralogs misaligning in polyploid species (Wallace and Mitchell, 2017). Samples with >50% missing data across all remaining sites were also filtered out.

The above filters reduced total missing data to <20% in each species (9% in kodo, 16.8% in little, 13% in proso). We tested imputation with LinkImpute (Money et al., 2015) to reduce this further; however, the imputed data did not result in any appreciable differences (Supplemental Fig. S1–S3) in downstream analyses, so all analyses were conducted with the unimputed data.

### Phylogeny

Phylogenetic networks were constructed using the NeighborNet method (Bryant and Moulton, 2003) in SplitsTree4 V4.14.4 (Huson and Bryant, 2005). Genotype data was converted to SplitsTree‐compatible NEXUS files by first using TASSEL version 5.2.29 (Bradbury et al., 2007) to export as a PHYLIP (interleaved) format, which was then converted to NEXUS format using Alter (Glez‐Peña et al., 2010) (http://www.sing‐group.org/ALTER/). The resulting files were manually edited to change the data type to dna and replace all colons (:) with underscores (_), at which point the files were loaded into SplitsTree for network creation.

### Population Structure Determination

Population structure analysis was performed with FastSTRUCTURE v1.0 (Raj et al., 2014), with the number of potential populations (*k*) varying from 1 to 15. The optimum population size was determined by chooseK and results were visualized with Distruct, both parts of the FastSTRUCTURE software package. Default parameters were used for all programs. Samples were assigned to a population if they had at least 60% membership in that population (this cutoff is arbitrary, but using a higher cutoff did not appreciably change population membership [data not shown]). Fixation index values were calculated for comparisons between all populations in each species as additional validation (Supplemental Data)

### Plotting Accessions by Geographic Data

Geographic data on kodo millet and little millet was plotted in Python using basemap (Hunter 2007) and the known GPS coordinates, then colored by subpopulation. Since proso millet had only the country of origin for some accessions, its map was created manually in Inkscape 0.91 (https://inkscape.org) by placing dots (colored by subpopulation) in the country of origin for each accession.

### Principal Coordinate Analysis

Genetic principal coordinates were calculated using multidimensional scaling analysis in TASSEL V5.2.29 (Bradbury et al., 2007).

### Heritability Analysis

Phenotype data for heritability analysis was taken from public data on the kodo and little millet core collections (Upadhyaya et al., 2014), and proso millet phenotype data was provided by collaborators at IRCISAT (Supplemental Data). Phenotypes were fit as part of a mixed linear model in TASSEL (Bradbury et al., 2007) with a kinship matrix as the only covariate using default parameters. Narrow‐sense heritability (*h* ^2^) was estimated as the ratio of genetic variance to total variance in the model.

### Software

The following software and packages were used as part of this analysis:

- SplitsTree4: V4.14.4 (Huson and Bryant, 2005)
- Faststructure: 1.0: https://rajanil.github.io/fastStructure/ (Raj et al., 2014)
- TASSEL 5.2.29: http://www.maizegenetics.net/tassel (Bradbury et al., 2007)
- PLINK 2.0: http://zzz.bwh.harvard.edu/plink/ (Purcell et al., 2007)
- Inkscape 0.91: https://inkscape.org/release/inkscape‐0.91/?latest=1
- GNU Parallel 20141022 (Tange, 2011)
- Matplotlib 2.2.2 (Hunter, 2007)
- Pandas 0.17 (McKinney 2010)
- NumPy 1.11.0 (Oliphant, 2006)
- Python 3.5.2
- VCF Tools 0.1.13 (Danecek et al., 2011)

## RESULTS AND DISCUSSION

### Genome‐Wide Marker Sets

Genotype data was generated using GBS (Elshire et al., 2011) with PstI restriction digestion. This resulted in ∼12,000 to 14,000 raw SNPs per species (Table 2), although many of these are probably artifacts resulting from misalignments. Low‐quality SNPs were removed by filtering the raw genotype data to remove sites with >50% missing data, minor allele frequency <5%, and >20 to 25% heterozygosity. (The heterozygosity filter removes false SNPs resulting from paralogs misaligning in polyploid species (Wallace and Mitchell, 2017)). Samples with >50% missing data across all remaining sites were also filtered out. The final, filtered genotype data (Supplemental Data) consists of 3461 SNPs across 165 accessions of kodo millet, 2245 SNPs across 165 accessions of little millet, and 1882 SNPs across 161 accessions of proso millet. (Table 2)

### Proso Millet

**Fig. 1:**
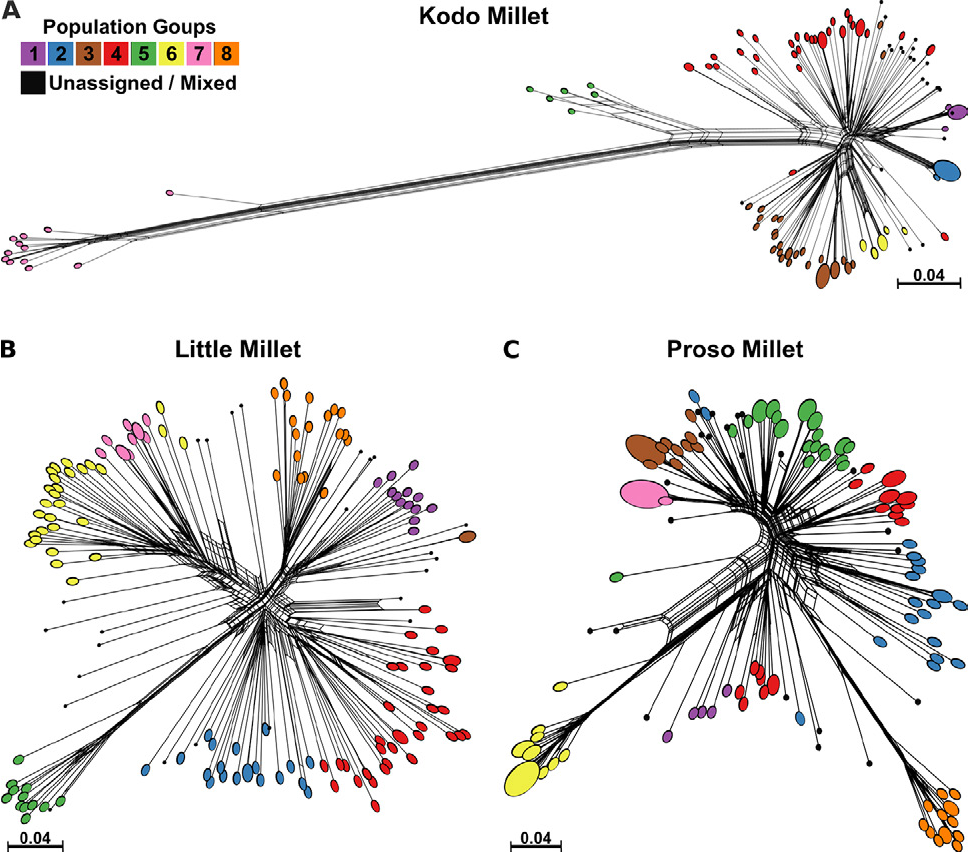
Phylogenetic trees for (A) kodo millet, (B) little millet, and (C) proso millet colored by population calls from fastSTRUCTURE for accessions that fit into a population with at least 60% fit. Each accession has one line that ends in a point and has branching to show alternative branching options. Where multiple points of the same population grouped closely together, a single oval with a size proportional to the number of accession it encompasses was used.

Population structure analysis of proso millet with fastSTRUCTURE (Raj et al., 2014) grouped the 161 postfiltering samples into eight putative subpopulations (Fig. 1). Some of the subpopulations consist almost entirely of pure individuals of that subpopulation, implying strong separation from the other subpopulations (e.g., Groups 7 and 8), while others (e.g., Groups 2–5) show significant admixture among the subpopulations. We observed that small changes in fastSTRUCTURE parameters would significantly alter the division and number of subpopulations in this admixed group (data not shown), further indicating that the divisions among the highly admixed subpopulations are weak and should be interpreted with caution.

**Table 2:** Number of single nucleotide polymorphisms (SNPs) and accessions before and after filtering.

Species	Raw SNPs	Filtered SNPs	Raw accessions	Filtered accessions
Kodo millet	12995	3461	190	165
Little millet	14473	2245	190	165
Proso Millet	12839	1882	190	161

**Fig. 2:**
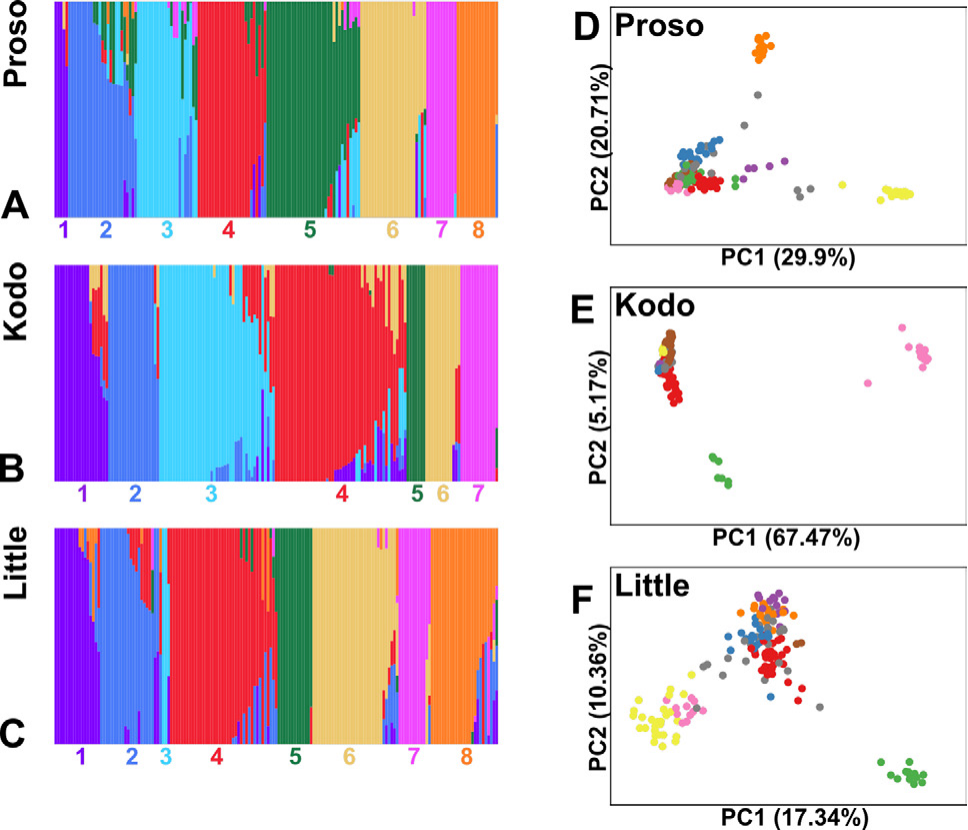
(A–C) Visual representation of how well individual accessions fit into putative populations from Distruct, a part of the fastSTRUCTURE program. The vertical bars each represent a single accession, and the colors correspond to populations as listed on the bottom of each subfigure: (A) proso millet, (B) kodo millet, and (C) little millet. (D–F) Principle coordinate analysis based off of genetic distances and colored by putative populations for (A) proso millet, (B) kodo millet, and (C) little millet.

A phylogenetic network of these samples (Fig. 1) mirrors this, where most of the subpopulations group together, although Subpopulations 6 and 8 are strongly separated from the rest. Divisions among the main group of populations appear weak, as evidenced by the large number of alternative splits (webbing) in the phylogenetic web and the fact that two populations (2 and 4) are split into two to three groups across the phylogeny. The genetic principal coordinates of these samples (Fig. 2) indicate a similar pattern, where most samples cluster together but with Subpopulations 6 and 8 distinct.

While the race data was not available for proso millet in this study, there is strong evidence that race is not a good indicator of genetic relatedness among accessions (Vetriventhan and Upadhyaya 2018). Existing core collections were designed with the races of the species being a central focus. Core collections likely could be improved by using population structure and genomic data to improve the genetic diversity of the collections.

The plotting of the 109 accessions with known geographic location supports the population analysis (Supplemental Fig. S4).

The reference genome was published after the bulk of the work for this paper had been done, and when analyses were reran using the reference genome, there were no significant difference found from the original analysis (Supplemental Fig. S5; the SNPs called from the new reference genome were included as well in Supplemental Data)

### Kodo Millet

Population structure analysis places the 165 kodo millet samples into seven putative subpopulations (Fig. 2). Most accessions cluster together, but two subpopulations (5 and 7) are strongly separated from the rest (Fig. 1,2). The separation of Subpopulation 7 from the other samples drives the largest genetic differences in this species, with 67.5% of the genetic variation along this axis (PC1 in Fig. 2).

These patterns of population structure do not correlate with existing race designations (Supplemental Fig. S6), implying that existing race designations do not strongly correlate to genetic groupings. Similar to proso millet, this implies that core collections of kodo millet could be improved by using population and genomic data instead of race designation.

Only 17 of the kodo millet accessions had known geographic origins, all of them in India. Plotting these origins geographically supports the population analysis by showing clustering of Population 4 (Supplemental Fig. S7), although more samples would be needed to completely confirm this interpretation.

### Little Millet

The 165 filtered accessions of little millet were grouped into eight putative subpopulations by fastSTRUCTURE (Fig. 2). Little millet appears to have weaker population structure than the other two species (compare the variance explained by each principal coordinate, Fig. 2). Subpopulation 5 is relatively well separated from the others, followed by Subpopulations 6 and 7, and then the other subpopulations being relatively close together. (These subpopulation designations would shift around with minor changes in fastSTRUCTURE parameters, implying they are very weakly separated [data not shown].) Similar to the other two species, little millet population structure did not correlate with existing race designations (Supplemental Fig. S8). Plotting the 29 accessions with known geographic location supports the population analysis by showing distinct clustering of Populations 4 and 5 (Supplemental Fig. S9).

### Validation of Genotype Data via Estimated Heritability

To validate these genetic data, we used public phenotype data on the kodo millet and little millet core collections (Upadhyaya et al.,2014) and proso millet phenotypic data that was provided by ICRISAT (Supplemental Data). These data were used to estimate narrow‐sense heritability (*h* ^2^) for flowering time and plant height (Table 3). Our expectation was that true genotype data should result in moderate to high heritability values for some phenotypes, especially ones (such as flowering time and plant height) that are known to have a strong genetic component.

Heritability was estimated using a mixed linear model in TASSEL (see Materials and Methods section) to obtain estimated variance components. Little millet traits exhibited heritability from 0.209 to 0.807, while kodo millet traits were slightly lower, ranging from 0.0 to 0.505 (Supplemental Data). There were no other phenotypes provided for proso millet other than what was included in Table 3. In all three species, flowering time and plant height were some of the most heritable traits (Table 3). These traits are all known to be under strong genetic control in other grass crops (e.g., Buckler et al., 2009; Peiffer et al., 2014; Mace et al., 2013; Morris et al., 2012; Ma et al., 2016; Alqudah and Schnurbusch, 2014). Since poor‐quality SNPs should show little to no relationship with phenotype, these results imply that the SNP datasets we have generated accurately represent the genetic variation within these populations and can be used for real‐world breeding applications.

**Table 3:** Estimated heritabilities for minor millets.

Millet species	Trait	Heritability
Little	Days to 50% flowering	0.807
Little	Plant height (cm)	0.664
Proso	Days to 50% flowering	0.69
Proso	Plant height (cm)	0.615
Kodo	Days to 50% flowering	0.505
Kodo	Plant height (cm)	0.293

## CONCLUSIONS

The results from this study represent one of the first genome‐wide analyses for proso millet and the first for kodo millet and little millet, three orphan crops that are important for food security in developing nations. We have identified thousands of SNPs in each of these species that accurately capture the population structure of each, as indicated by the geographic correlations and estimates of narrow‐sense heritability. Our analyses can be used as a foundation for further exploration into the genetics of these species including selecting appropriate breeding materials and identifying priority populations for further collection and curation.

Existing core collections were designed with the races of the species being a central focus. The evidence strongly implies that race is not a good determiner of genetic relatedness, and, as such, the core collections likely could be improved by using modern genomic data to improve the genetic diversity of the collections.

For both major and minor crops, obtaining genetic data is now (almost) trivial, even in species with highly complex genomes and no prior history of genetic analysis. As the price of DNA sequencing continues to drop, more and more orphan species will have genotype data available. The major question going forward will be how to best deploy these data to benefit breeders and growers. Given that the price of phenotyping is often the limiting factor in many studies (Cobb et al., 2013), finding ways to deploy genomic prediction and high‐throughput phenotyping for orphan crops likely will be the next major step to democratize modern genomics for the developing world.

### Supplemental Information

Supplemental Fig. S1. Principal coordinate analysis for kodo millet using imputed genotype data. Population structure and groupings are almost identical to those made with unimputed data (main text Fig. 2E).

Supplemental Fig. S2. Principal coordinate analysis for little millet using imputed genotype data. Population structure and groupings are almost identical to those made with unimputed data (main text Fig. 2D).

Supplemental Fig. S3. Principal coordinate analysis for kodo millet using imputed genotype data. Population structure and groupings are almost identical to those made with unimputed data (main text Fig. 2F).

Supplemental Fig. S4. Plotting of Proso millet accessions by country of origin. Within‐country coordinates were not available, so points within each country are displayed as a grid. Points are colored by subpopulation (see main text Fig. 2); black indicates the accession didn't fit into any given population.

Supplemental Fig. S5. Principal Coordinate analysis of Proso millet using the recently published reference genome (Zou et al., 2019). These results are consistent with those done without a reference genome (main text Fig. 2D).

Supplemental Fig. S6. PC analysis of kodo millet using race to color the accessions. Races do not correspond with genetic clusters, indicating that they do not reflect genetic relationships

Supplemental Fig. S7. Kodo millet accessions plotted by GPS coordinates. Only a few kodo millet accessions had GPS data available, though those that do are relatively consistent with subpopulations clustering by region of origin. Points are colored by subpopulation (see main text Fig. 2); black indicates the accession didn't fit into any given population.

Supplemental Fig. S8. PC analysis of Little millet using race to color the accessions. Race does not show the diversity of genetic clusters, and as such it is not a good reflection of genetic relationships.

Supplemental Fig. S9. Little millet accessions plotted by GPS coordinates. Only a few little millet accessions had GPS data available, though those that do are relatively consistent with subpopulations clustering by region of origin. Points are colored by subpopulation (see main text Fig. 2); black indicates the accession didn't fit into any given population.

### Author Contributions

Santosh Deshpande, Mani Vetriventhan, and Hari Upadhyaya grew the plants and extracted the DNA. Jason Wallace called the SNPs. Matthew Johnson cleaned and analyzed the data. Matthew Johnson wrote the first draft of the paper and all authors contributed to edits.

### Conflicts of Interest

The authors declare that they have no known conflicts of interests.

## Supporting information

Supplemental File 1
