# Supplementary material for: Genome‐Wide Population Structure Analyses of Three Minor Millets: Kodo Millet, Little Millet, and Proso Millet
Source: Plant Genome. 2019 Nov 1;12(3):190021. doi: 10.3835/plantgenome2019.03.0021 (PMC12810065; doi:10.3835/plantgenome2019.03.0021)
Supplement: Supplementary file 1 [file TPG2-12-190021-s001.pdf]

Supplemental Figures for Small Millet Paper

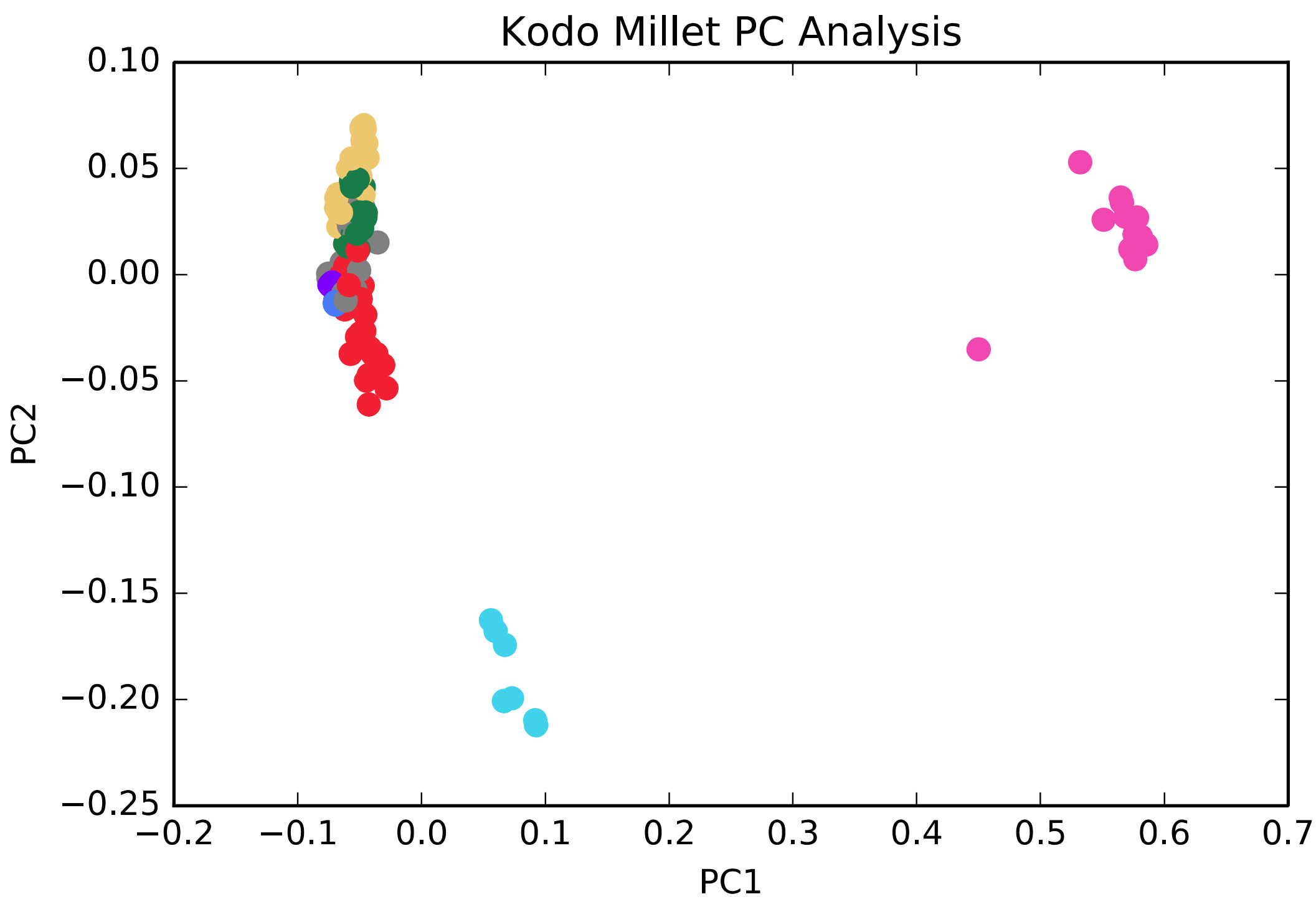

Supplemental Figure 1
Principal coordinate analysis for kodo millet using imputed genotype data. Population structure and groupings are almost identical to those made with unimputed data (main text Figure 2E).

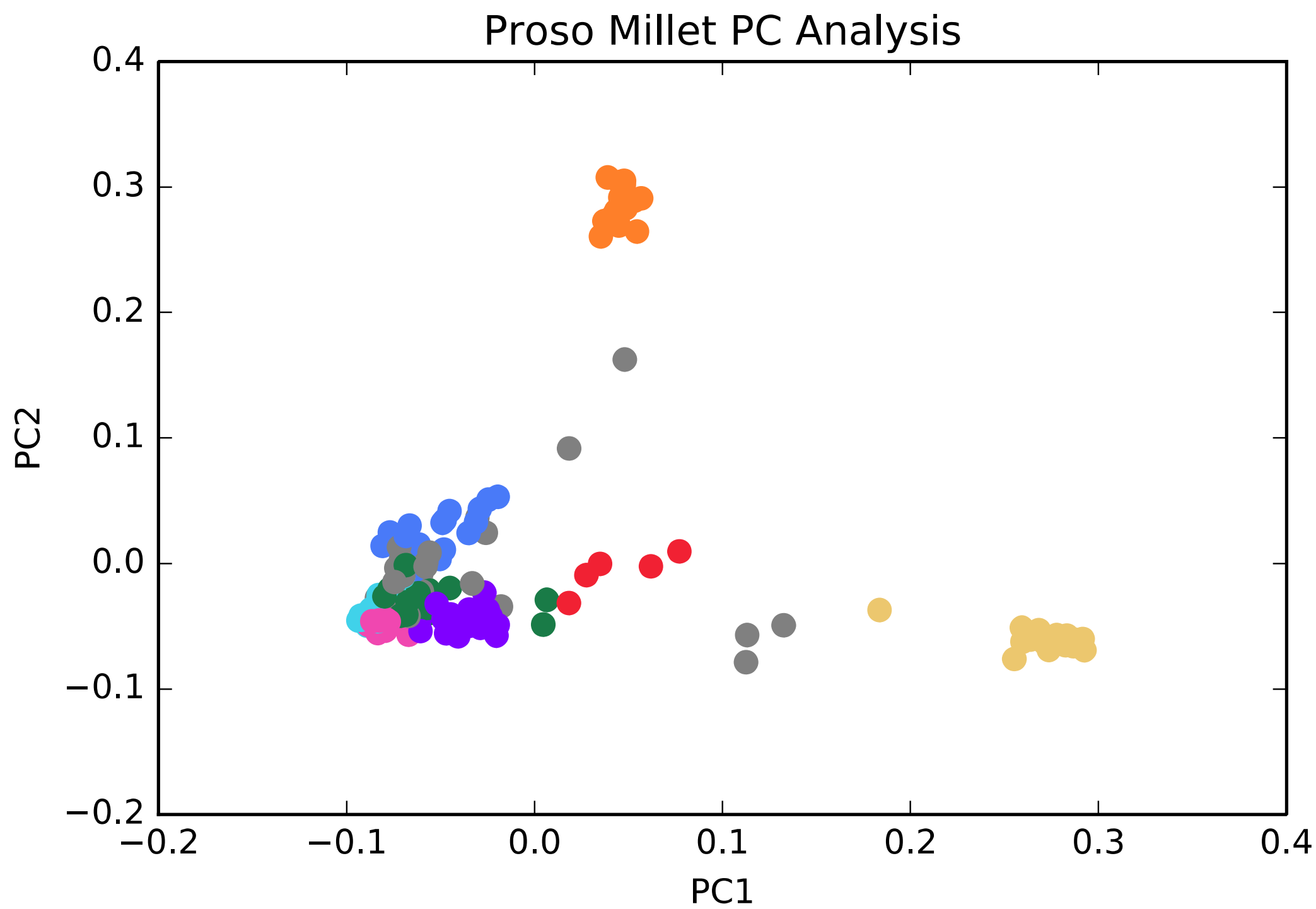

Supplemental Figure 2
Principal coordinate analysis for little millet using imputed genotype data. Population structure and groupings are almost identical to those made with unimputed data (main text Figure 2D).

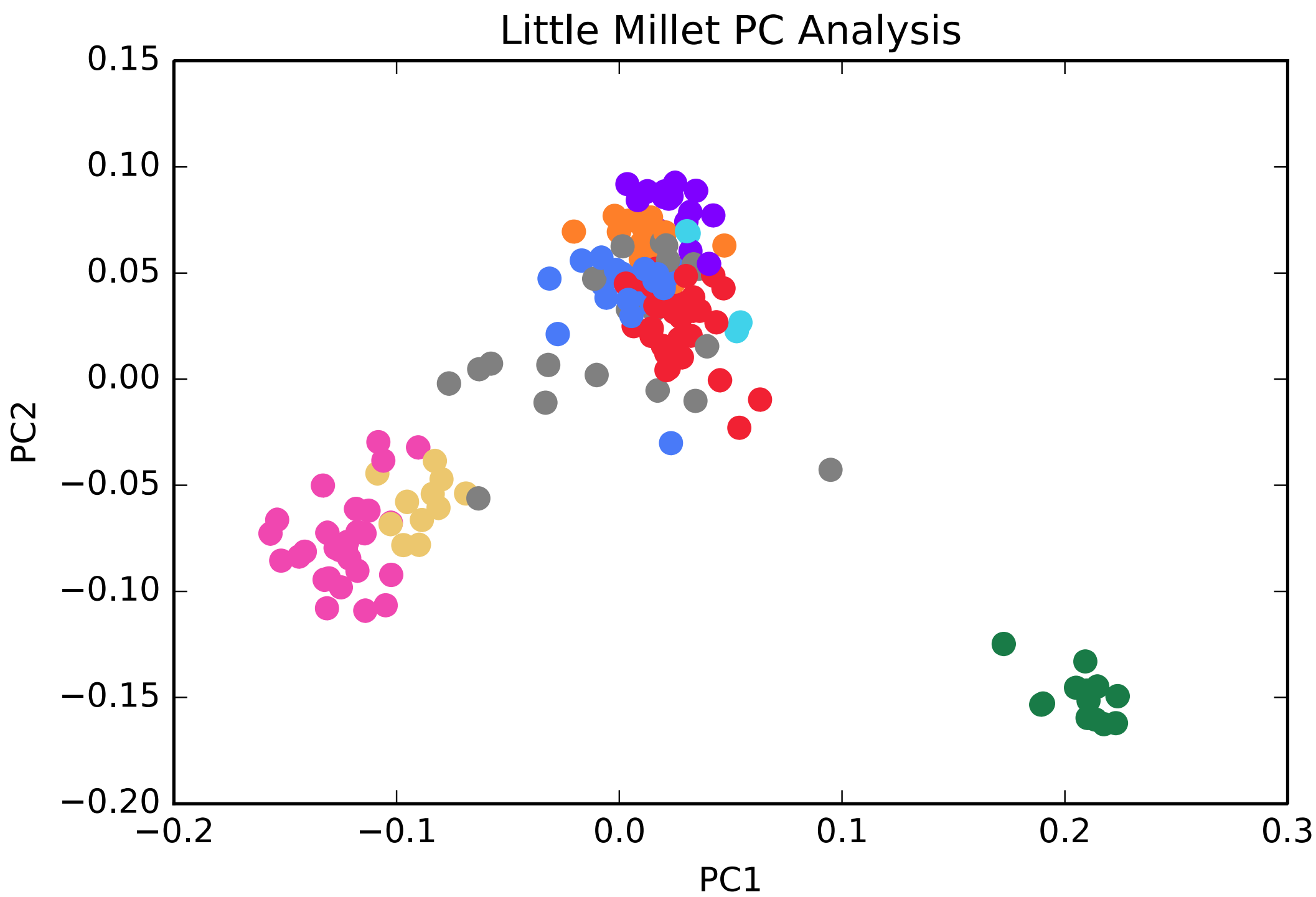

Supplemental Figure 3
Principal coordinate analysis for kodo millet using imputed genotype data. Population structure and groupings are almost identical to those made with unimputed data (main text Figure 2F).

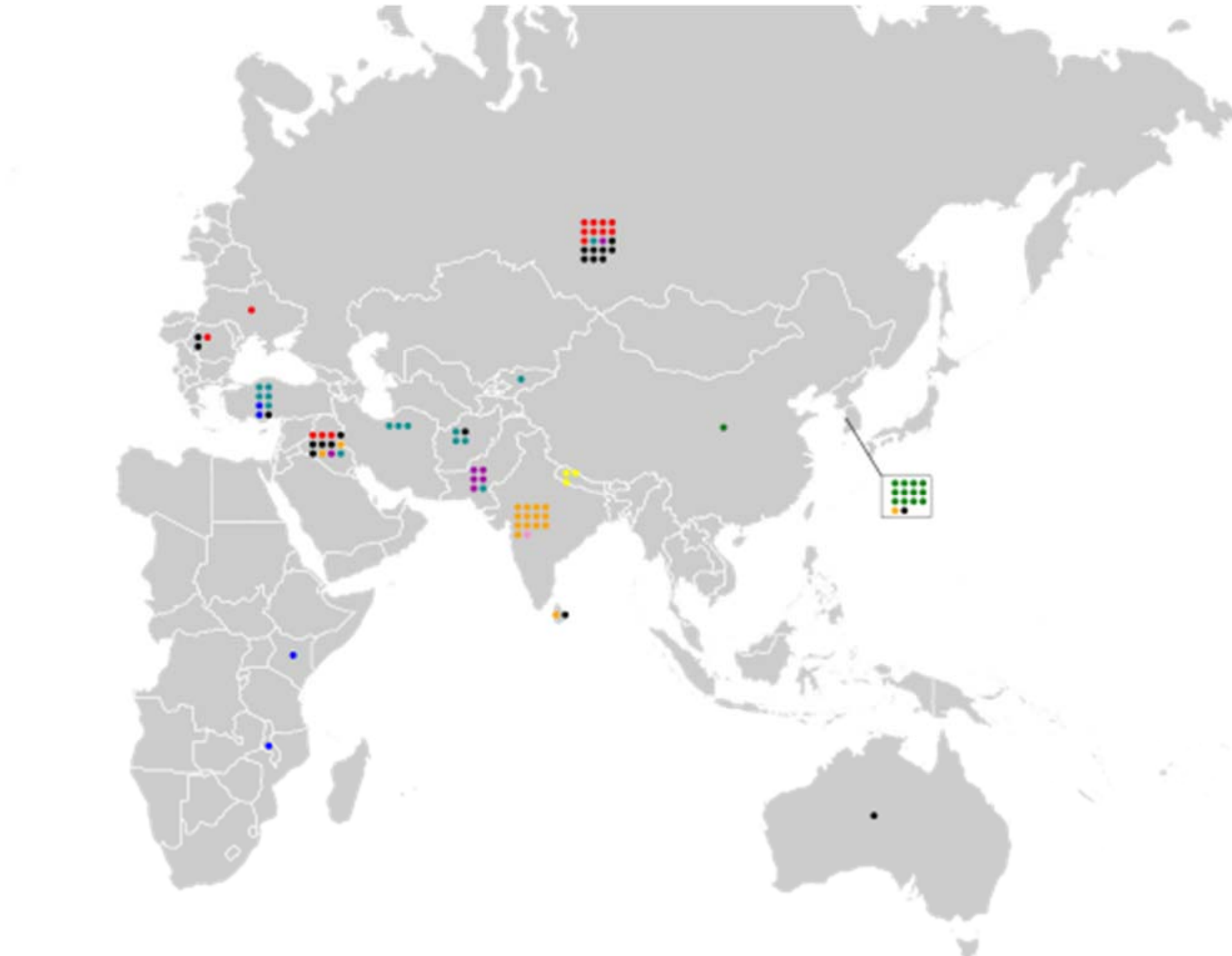

Supplemental Figure 4

Plotting of Proso millet accessions by country of origin. Within-country coordinates were not available, so points within each country are displayed as a grid. Points are colored by subpopulation (see main text Figure 2); black indicates the accession didn't fit into any given population.

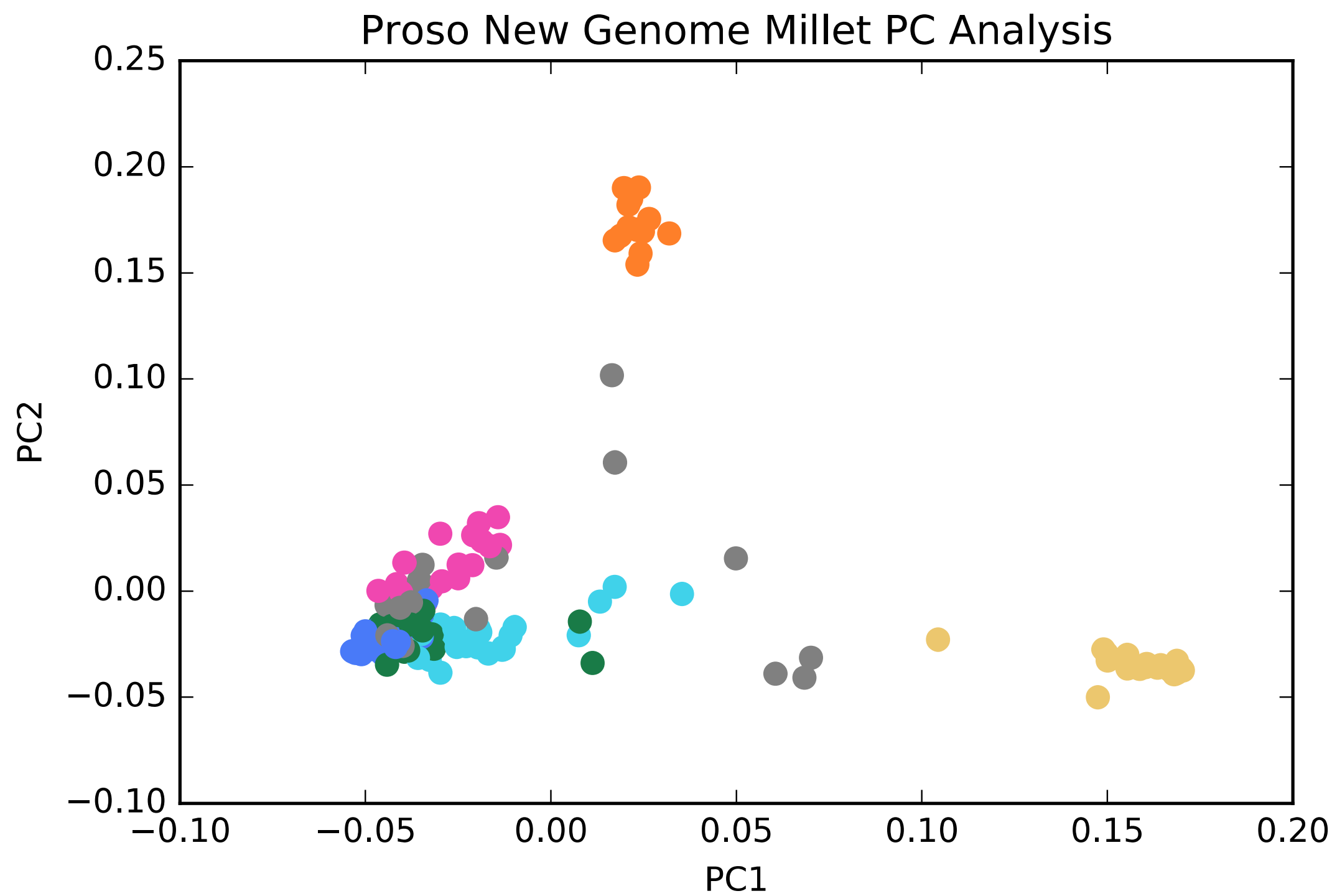

Supplemental Figure 5

Principal Coordinate analysis of Proso millet using the recently published reference genome (Zou et al., 2019). These results are consistent with those done without a reference genome (main text Figure 2D).

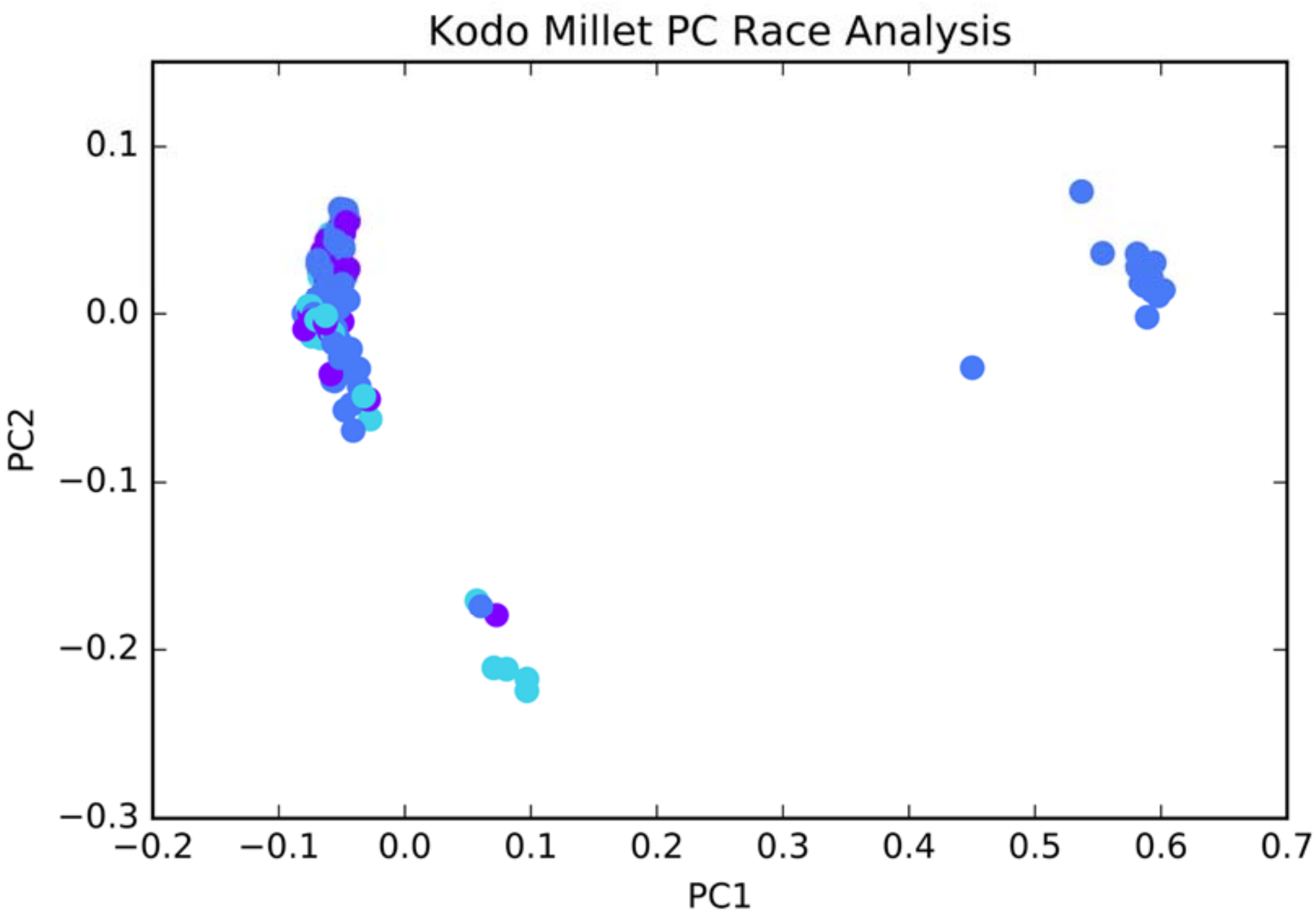

Supplemental Figure 6
PC analysis of kodo millet using race to color the accessions. Races do not correspond with genetic clusters, indicating that they do not reflect genetic relationships.

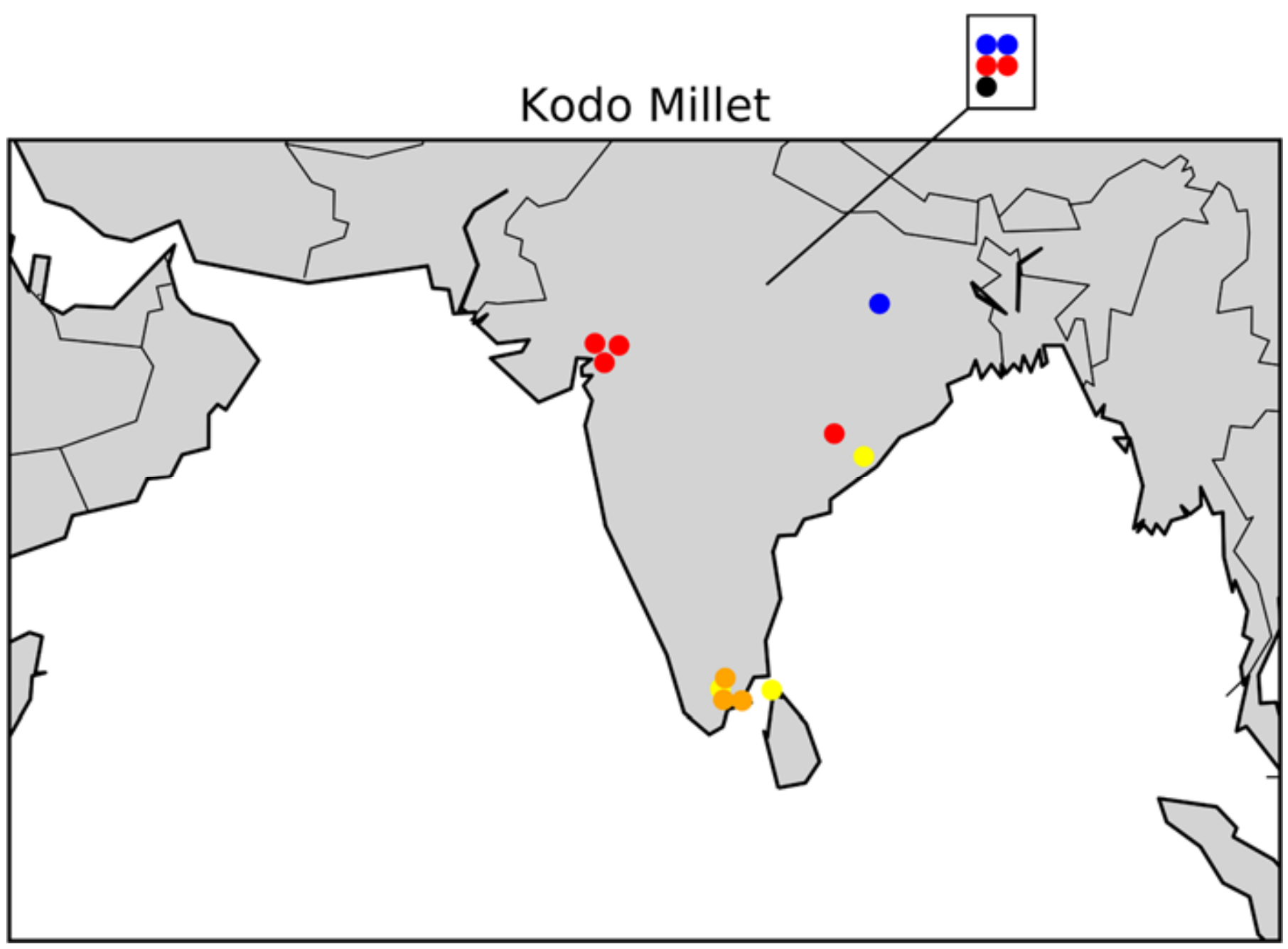

Supplemental Figure 7
Kodo millet accessions plotted by GPS coordinates. Only a few kodo millet accessions had GPS data available, though those that do are relatively consistent with subpopulations clustering by

region of origin. Points are colored by subpopulation (see main text Figure 2); black indicates the accession didn't fit into any given population.

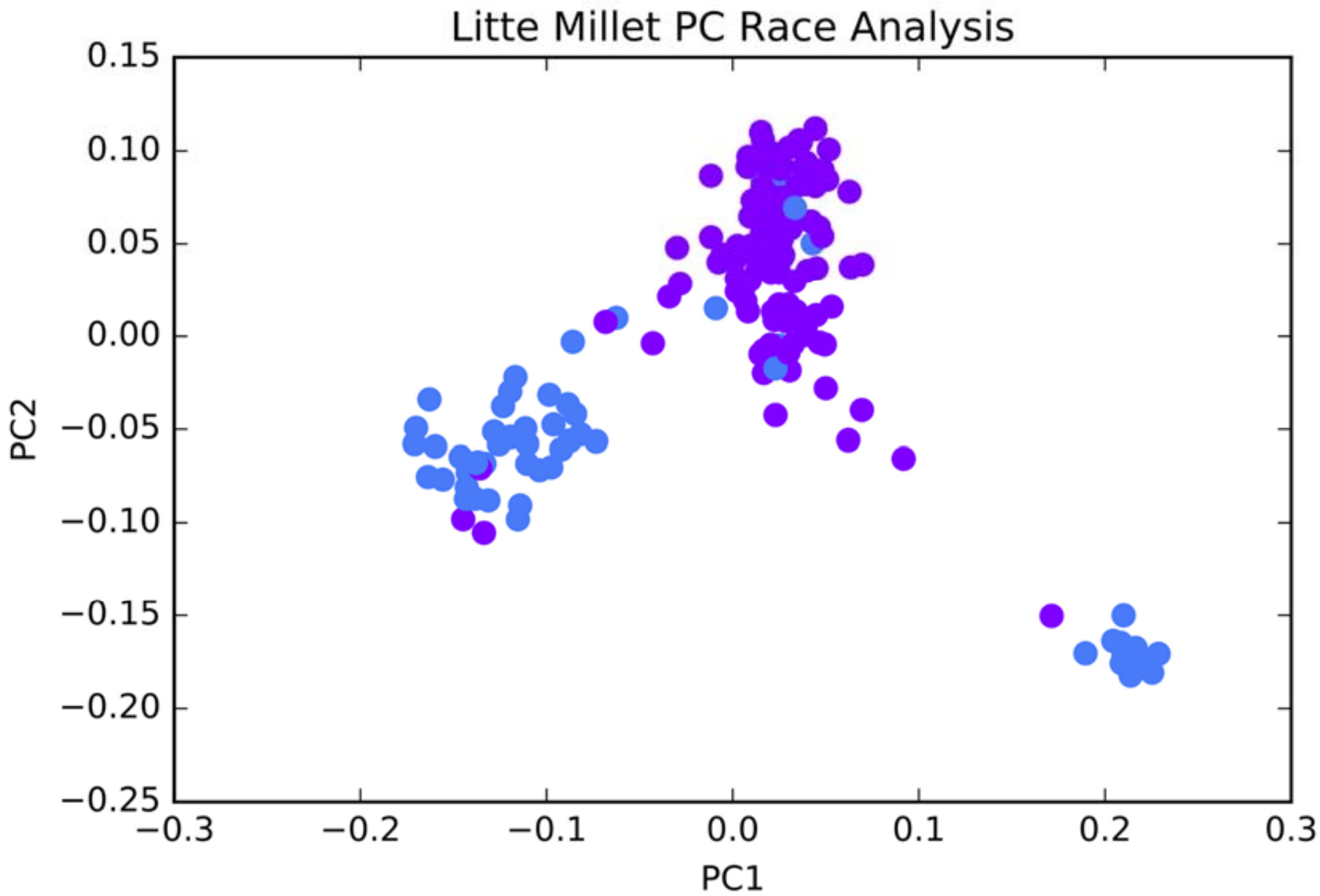

Supplemental Figure 8
PC analysis of Little millet using race to color the accessions. Race does not show the diversity of genetic clusters, and as such it is not a good reflection of genetic relationships.

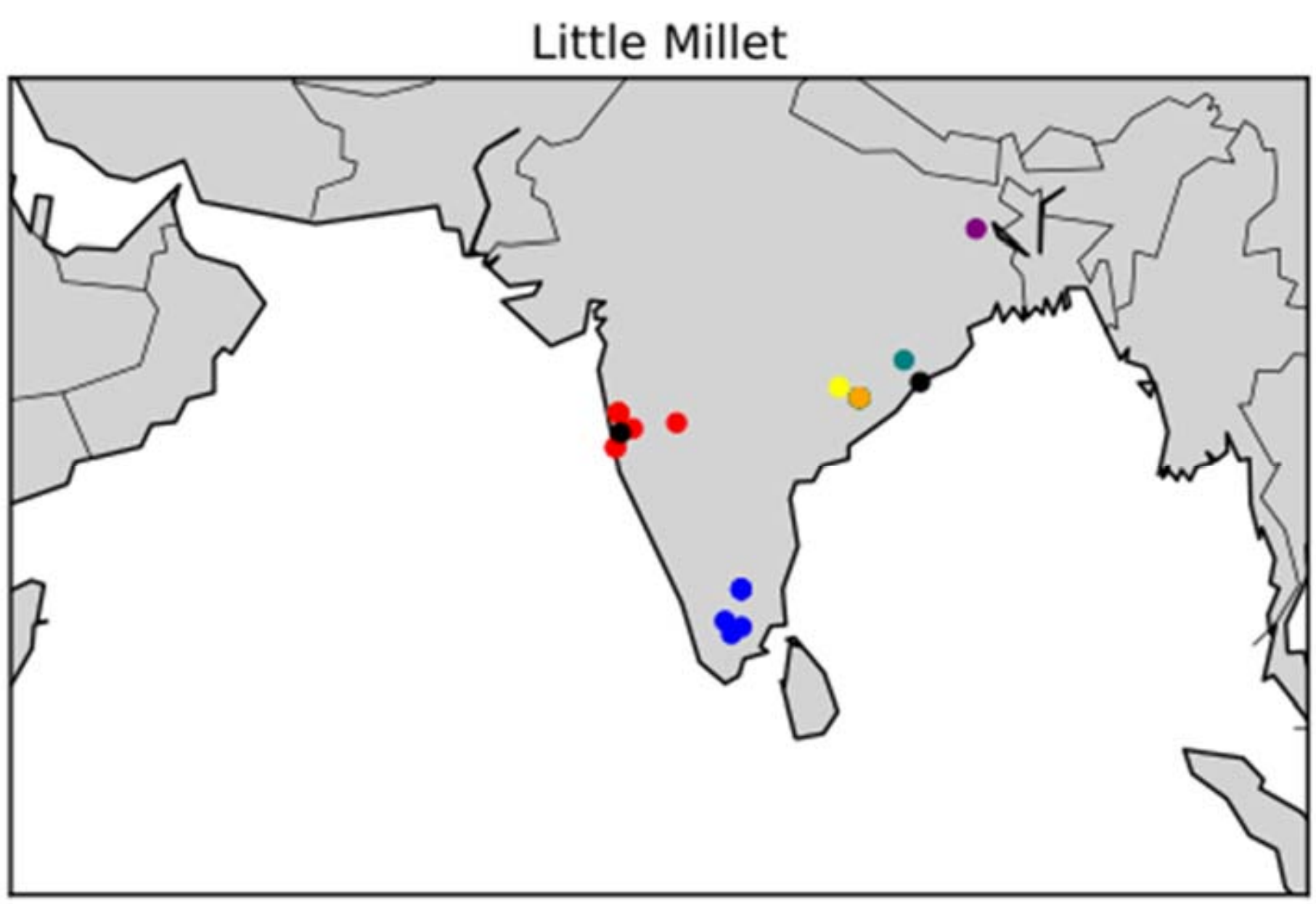

Supplemental Figure 9
Little millet accessions plotted by GPS coordinates. Only a few little millet accessions had GPS data available, though those that do are relatively consistent with subpopulations clustering by region of origin. Points are colored by subpopulation (see main text Figure 2); black indicates the accession didn’t fit into any given population.
